# Abcès thyroïdien révélant un basedow: à propos d’un cas et revue de la littérature

**DOI:** 10.11604/pamj.2016.24.204.9902

**Published:** 2016-07-08

**Authors:** Meriem Chenguir, Hajar Souldi, Fatima Zahra Loufad, Sami Rouadi, Reda Abada, Mohamed Roubal, Mohamed Mahtar

**Affiliations:** 1 Service d’ORL et de Chirurgie Cervico-Faciale, Hôpital 20 Août, Casablanca, Maroc

**Keywords:** Goitre toxique, abcès thyroïdien, thyroïdite, Toxic goiter, thyroid abscess, thyroiditis

## Abstract

L’abcès thyroïdien est une entité clinique très rare. Elle représente 0,1% des pathologies chirurgicales de la thyroïde. Les caractéristiques anatomique et physiologique de la glande lui procurent une résistance vis-à-vis des agents pathogènes. Les infections à Streptococcies et Staphylococcus sont les plus fréquentes. La tuberculose reste rarement rapportée dans la littérature. La survenue de l’infection sur goitre toxique est exceptionnelle. Les auteurs rapportent un cas rare d’abcès thyroïdien révélant un goitre toxique chez un jeune de 22 ans. Il s’est présenté aux urgences ORL pour une tuméfaction cervicale antérieure, légèrement latéralisée à gauche, mobile à la déglutition, associée à des hémoptysies, signes de dysthyroïdie, fièvre et sueurs nocturnes. La TDM cervicale a montré une masse occupant le lobe thyroïdien gauche de contenu liquidien mesurant 2 cm, avec un liquide purulent à la cytoponction. L’examen cyto-bactériologique a mis en évidence un Staphylococcus avec recherche de BK positive. Une radiographie thoracique a été demandée montrant un foyer alvéolaire pulmonaire apical droit. L’étude cytobactériologique des crachats ont isolé le bacille de koch. Le bilan biologique thyroïdien était en faveur d’un Basedow. La prise en charge était médicale comprenait une tri-antibiothérapie par voie parentérale, anti-bacillaire et anti-thyroïdien de synthèse avec bonne évolution. Le diagnostic de la tuberculose doit être évoqué devant toute abcédation thyroïdienne avec un tableau clinique peu bruyant. Il s’agit le plus souvent d’une dissémination hématogène à partir d’un autre foyer de primo-infection surtout pulmonaire. Le traitement est basé sur les anti-bacillaires associé parfois à la chirurgie.

## Abstract (en)

Thyroid abscess is a very rare clinical entity. It accounts for 0.1% of the surgical pathologies of the thyroid gland. The anatomical and physiological characteristics of the gland give it resistance to pathogens. Streptococcal and Staphylococcal are the most common. Tuberculosis is rarely reported in the literature. The occurrence of infection associated with toxic goiter is exceptional. The authors report a rare case of a 22-year-old young patient with thyroid abscess revealing a toxic goitre. He presented to our otorhinolaryngology emergency unit with anterior cervical swelling, slightly lateralized to the left, moving on swallowing, associated with hemoptysis, signs of thyroid dysfunction, fever, night sweats. Cervical CT scan showed a mass occupying the left lobe of the thyroid gland, with fluid content, measuring 2 cm and with purulent fluid collected via fine needle aspiration biopsy. Cytobacteriological examination showed Staphylococcus with positive BK test. Patient underwent chest radiograph showing right apical pulmonary alveolar. Cytobacteriological examination of sputum isolated Koch bacillus. Thyroid biological assessment was in favor of Graves-Basedow disease. The management was medical and included parenteral triple antibiotic, anti-bacillary and anti-thyroid synthesis therapy with good evolution. The diagnosis of tuberculosis should be suspected in patients with thyroid abscess formation associated with an unclear clinical picture. This is most often caused by hematogenous spread from another primary infection, particularly a pulmonary infection. The treatment is based on antibacillary drugs sometimes associated with surgery.

## Introduction

L’abcès de la thyroïde est une entité pathologique rare représentant seulement 0.1% des pathologies chirurgicales de la thyroïde. Les caractéristiques anatomique et physiologique de cette glande thyroïde lui confèrent une résistance vis à vis des germes pathogènes. La survenue de cette entité pathologique impose la présence de facteur de risque induisant une propagation de l’infection à la glande thyroïde en altérant sa résistance. Le principal facteur de risque est l’altération congénitale de la morphologie de la glande (fistule du sinus piriforme) communiquant ainsi la loge thyroïdienne au pharynx. Chez l’adulte est souvent une dissémination à partir d’un foyer infectieux de proximité ou à distance (bactériémie). D’autres facteurs induisant l’infection de la thyroïde comprennent: une modification de la morphologie thyroïdienne, immunodépression ou facteur iatrogène. Les infections à Streptococcies sp et Staphylococcus sp sont les plus fréquentes et représentent 70% des cas. La tuberculose reste rarement rapportée dans la littérature. Les auteurs rapportent un cas rare d’abcès thyroïdien révélant une maladie de Basedow chez un jeune de 22 ans. Le diagnostique de tuberculose est retenue devant la culture du liquide de cyto-ponction. La dissémination est hématogène à partir d’un foyer de primo-infection pulmonaire.

## Patient et observation

**Figure 1:**
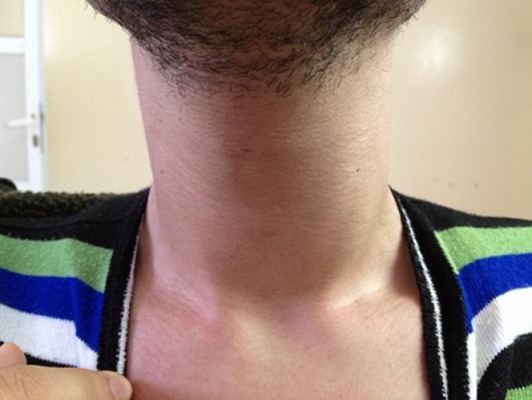
Légère tuméfaction cervicale anterieure

**Figure 2:**
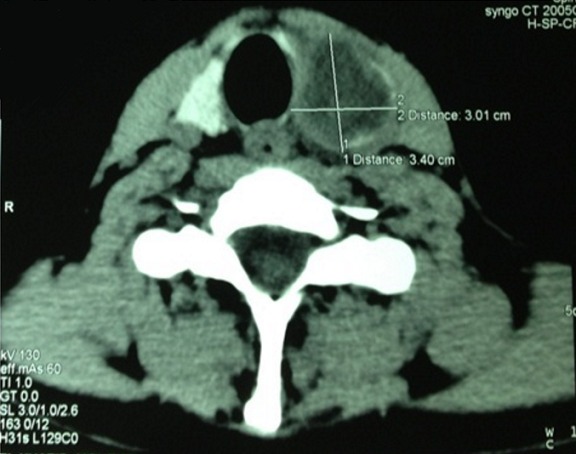
Scanner cervical montrant une masse liquidienne occupant le lobe thyroïdien gauche

**Figure 3:**
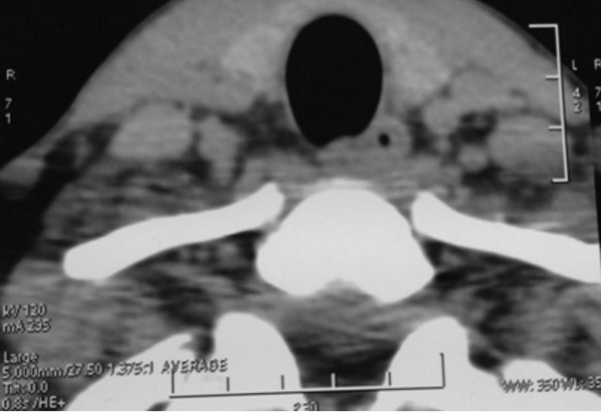
TDM cervicale de contrôle montrant un tarissement de l’abcès

Les auteurs rapportent un cas rare d’abcès thyroïdien, chez un jeune de 22 ans sans antécédents pathologiques particuliers, qui s’est présenté aux urgences ORL pour une tuméfaction cervicale antérieure évoluant depuis 15 jours. La symptomatologie est associée à des palpitations, nervosité, tremblement des extrémités, hémoptysies minimes avec une fièvre et sueurs nocturnes. L’examen clinique montre un patient fébrile à 39°C, tachycarde, eupneïque, avec présence d’une légère tuméfaction basi-cervicale antérieure, douloureuse à la palpation, de 3cm du grand axe, légèrement latéralisée à gauche, mobile à la déglutition sans ADP cervicales palpables (Figure 1). Le reste de l ‘examen ORL est sans particularité. Le bilan biologique réalisé, objective des perturbations biologiques: anémie hypochrome microcytaire, hyperleucocytose, VS et CRP élevées avec augmentation de T4 libre et TSH basse. L’échographie et la TDM cervicale faites en urgence, montrent une masse occupant le lobe thyroïdien gauche de contenu liquidien mesurant 34x27 cm avec un aspect hétérogène du reste du parenchyme thyroïdien (Figure 2). La radiographie thoracique objective un foyer alvéolaire pulmonaire apical droit. Le diagnostic d’abcès de la thyroïde était évoqué, à côté d’une tumeur thyroïdienne nécrosée. L’origine tuberculeuse a était aussi discutée devant l’image thoracique. La cytoponction à l ´aiguille a ramenée 2 ml de liquide purulent, ce qui a permis de confirmer le diagnostic d’abcès thyroïdien. Une tri-antibiothérapie parentérale probabiliste a été démarrée secondairement adaptée à l’antibiogramme. L’étude cyto-bactériologique du liquide de cyto-ponction a mis en évidence un Staphylococcus. La bacille de koch était identifiée après culture aussi bien dans le liquide de cytoponction qu’au niveau des sécrétions respiratoires. L’évolution était bonne sous tri-antibiothérapie parentérale pendant 48h avec relais per-os après une nette réduction de l’abcès au scanner de contrôle. Un traitement anti-bacillaire a été démarré après confirmation de diagnostic de tuberculose pulmonaire et thyroïdienne. Le bilan complémentaire immunitaire thyroïdien était en faveur de maladie de basedow pour lequel le patient a été mis sous antithyroïdien de synthèse. Après 6 mois de traitement anti-bacillaire, le bilan radiologique de contrôle a objectivé un nettoyage du foyer pulmonaire et tarissement complet de l’abcès thyroïdien (Figure 3).

## Discussion

L’abcès de la thyroïde est une entité pathologique rare représentant seulement 0,1% des pathologies chirurgicales de la thyroïde [1]. Ceci est attribué à sa localisation anatomique isolée grâce à sa capsule, sa vascularisation largement anastomosée et sa concentration en iode [2]. La survenue de cette entité pathologique impose la présence de facteur de risque induisant une propagation de l’infection à la glande thyroïde en altérant sa résistance. L’abcès de la thyroïde touche l’enfant plus que l’adulte. Ceci est du à l’existence de malformation congénitale telles les fistules du tractus thyréoglosse et fistules du 4ème poche endobranchiale communiquant ainsi la loge thyroïdienne à l’hypopharynx [1]. Chez l’adulte, multiple étiologies sont en cause. Une infection peut résulter soit par inoculation iatrogène d’un corps étranger, tels que la cytoponction thyroïdienne, une perforation de l’œsophage ou de l’hypo pharynx, soit par dissémination sanguine ou lymphatique à partir d’un foyer à distance. D’autres facteurs induisant l’infection de la thyroïde comprennent: une modification de la morphologie thyroïdienne, immunodépression [3, 4]. Les infections à Streptococcies et Staphylococcus sont les plus fréquentes et représentent 70% des cas [5]. La tuberculose reste rarement rapportée dans la littérature. Elle représente 0,1 à 0,4% de toutes les localisations [6, 7]. L’existence de modification thyroïdienne avec des troubles vasculaires pourraient constituer un facteur de sensibilisation à l’atteinte bacillaire (GURKAN) [8]. L’infection de la glande peut être secondaire lors d’une bacillémie tuberculeuse survenant au cours d’une primo-infection, comme le cas de notre patient ou exceptionnellement primitive. La tuberculose thyroïdienne survient à tout âge avec cependant une prédominance de la tranche d’âge 20-50 ans. Le sexe féminin semble le plus touché (70 à 80%), en rapport avec la fréquence de la pathologie thyroïdienne chez la femme [9]. Le tableau clinique n’est pas spécifique. Diverses manifestations peuvent se rencontrer évoluant dans un mode chronique ou subaigu: une tuméfaction cervicale isolée ou associée à des adénopathies cervicale, réalisant un syndrome pseudotumoral. On peut observer aussi une modification d’un goitre préexistant ou tableau de thyroïdite [10]. Très rarement se présente un abcès de la glande thyroïde comme notre cas.

Parfois la tuberculose thyroïdienne se manifeste par des formes compliquées telles une fistulisation à la peau d’un abcès froid, compression de l’axe aérodigestif, compression nerveuse récurentielle voir même sympathique [3, 11]. Les bilans fonctionnels thyroïdiens sont normaux chez la plupart des patients. Une thyréotoxicose ou une hypothyroïdie peut être exceptionnellement observée. L’hyperthyroïdie peut s’installer au début suite à une destruction de la glande et à la libération massive des hormones thyroïdiennes. Par la suite une hypothyroïdie peut survenir par destruction totale du parenchyme [12]. L’association maladie de basedow et tuberculose thyroïdienne, comme pour notre cas, n ‘a jamais été décrit. L’existence d’un foyer tuberculeux concomitant ou séquellaire permet d’évoquer le diagnostic et réaliser un bilan tuberculeux. L’aspect à l’imagerie n’est pas spécifique. Toutefois, la présence au scanner d’une lésion, à paroi épaisse prenant fortement le contraste avec de la nécrose au centre, est caractéristique de la tuberculose. Le piège est de penser immédiatement au cancer qui présente le principal diagnostic différentiel. Alors que la démarche diagnostique devrait comporter un bilan tuberculeux préalable. La radiographie thoracique peut montrer un foyer concomitant ou séquellaire. Le diagnostic positive de tuberculose repose soit sur la cytoponction à l’aiguille, suivie d’une étude cyto-bactériologique et même d’une PCR mettant en évidence le bacille acido-alcoolo-résistant (BAAR), soit histologique sur pièce anapathologique après chirurgie. La cytoponction n‘a de valeur que si elle est positive [10].

En effet, la chirurgie, suivie de l’examen anatomopathologie peut être le seul remède pour confirmer le diagnostic. Le traitement de la tuberculose thyroïdienne est médical si un diagnostic positif au préalable a été retenu, et consiste en l’association de puissants antibacillaires. Le traitement chirurgical n’est indiqué que dans des formes anatomiques particulières telles que l’abcès ou lorsque le diagnostic oriente vers une pathologie tumorale. Dans notre cas le traitement médical était suffisant vue la taille de la collection et le tarissement de l’abcès après traitement anti-bacillaire.

## Conclusion

La tuberculose thyroïdienne est une affection rare dont le diagnostic est difficile. Seul l’examen bactériologique et/ou histologique permet d’affirmer la nature bacillaire de l’atteinte thyroïdienne. Le traitement est médical et éventuellement chirurgical en fonction de la forme anatomique.
